# IDTAXA: a novel approach for accurate taxonomic classification of microbiome sequences

**DOI:** 10.1186/s40168-018-0521-5

**Published:** 2018-08-09

**Authors:** Adithya Murali, Aniruddha Bhargava, Erik S. Wright

**Affiliations:** 1 Department of Computer Sciences, University of Wisconsin-Madison, Madison, WI 53715 USA; 2 Department of Electrical and Computer Engineering, University of Wisconsin-Madison, Madison, WI 53715 USA; 3 Department of Biomedical Informatics, Pittsburgh Center for Evolutionary Biology and Medicine, School of Medicine, University of Pittsburgh, 426 Bridgeside Point II, 450 Technology Dr, Pittsburgh, PA 15219 USA

**Keywords:** Microbiome, 16S rRNA gene sequencing, ITS sequencing, Classification, Taxonomic assignment, Reference taxonomy

## Abstract

**Background:**

Microbiome studies often involve sequencing a marker gene to identify the microorganisms in samples of interest. Sequence classification is a critical component of this process, whereby sequences are assigned to a reference taxonomy containing known sequence representatives of many microbial groups. Previous studies have shown that existing classification programs often assign sequences to reference groups even if they belong to novel taxonomic groups that are absent from the reference taxonomy. This high rate of “over classification” is particularly detrimental in microbiome studies because reference taxonomies are far from comprehensive.

**Results:**

Here, we introduce IDTAXA, a novel approach to taxonomic classification that employs principles from machine learning to reduce over classification errors. Using multiple reference taxonomies, we demonstrate that IDTAXA has higher accuracy than popular classifiers such as BLAST, MAPSeq, QIIME, SINTAX, SPINGO, and the RDP Classifier. Similarly, IDTAXA yields far fewer over classifications on Illumina mock microbial community data when the expected taxa are absent from the training set. Furthermore, IDTAXA offers many practical advantages over other classifiers, such as maintaining low error rates across varying input sequence lengths and withholding classifications from input sequences composed of random nucleotides or repeats.

**Conclusions:**

IDTAXA’s classifications may lead to different conclusions in microbiome studies because of the substantially reduced number of taxa that are incorrectly identified through over classification. Although misclassification error is relatively minor, we believe that many remaining misclassifications are likely caused by errors in the reference taxonomy. We describe how IDTAXA is able to identify many putative mislabeling errors in reference taxonomies, enabling training sets to be automatically corrected by eliminating spurious sequences. IDTAXA is part of the DECIPHER package for the R programming language, available through the Bioconductor repository or accessible online (http://DECIPHER.codes).

**Electronic supplementary material:**

The online version of this article (10.1186/s40168-018-0521-5) contains supplementary material, which is available to authorized users.

## Background

It has become increasingly clear that the microbiome is critically important to human and ecosystem health [1]. Microbiome studies frequently involve sequencing a taxonomic marker, such as the 16S ribosomal RNA (rRNA) gene or internal transcribed spacer (ITS), to identify the microorganisms that are present in a sample of interest. These sequences can then be classified into a taxonomic group, which facilitates comparing across studies and acquiring additional information about the microorganisms. Classification relies on a training set containing sequence representatives belonging to known microbial taxa. Since only a fraction of microbial taxa have been characterized, it is anticipated that a large number of microorganisms from many environments belong to taxonomic groups that are unrepresented in the training set [2–4]. Thus, the objective of taxonomic classification is to accurately assign query sequences to their respective group in the reference taxonomy, while avoiding the assignment of sequences belonging to novel groups that are absent from the training set.

A major challenge to classification is that there is no standard definition of what constitutes a taxonomic group (e.g., genus or species) of microorganisms. Although there are many exceptions, strains belonging to the same genus tend to have about 95% or greater similarity in 16S rRNA gene sequence. Therefore, a common classification approach is simply to label a sequence based on its nearest neighbor in a training set using a tool such as BLAST [5]. Sequences are left unlabeled, or assigned to a higher rank (e.g., family), when they are not within a specified distance (e.g., 5%) of any reference sequence. Nearest neighbor methods are popular in part due to their simplicity and clearly defined basis for taxonomic assignment, but frequently fail where taxonomic groups do not conform to standard distance cutoffs [6].

Phylogenetic-based approaches are similar to nearest neighbor methods but use a phylogenetic framework for determining neighbors. Unlike sequence identity, phylogenetics can account for variation in evolutionary rates across sites and other details of sequence evolution. Capitalizing on the fact that taxa in reference taxonomies are often delineated using a phylogenetic tree, a number of different phylogenetic-based methods have been proposed [7–9]. These methods use a variety of approaches for calculating their confidence in taxonomic assignments, that is, how to best determine whether a new leaf of the tree belongs to any of the taxonomic groups that surround it on the tree. As in the case of distance-based approaches, it is often unclear whether a new leaf of the tree represents a novel taxon or an extension of an existing group.

In principle, machine learning is highly amenable to “learning” variable definitions of what constitutes a taxonomic group across the tree of life. The most popular machine learning approach for taxonomic classification is the naïve Bayes method used by the RDP Classifier [6], which has been implemented in popular microbiome software such as mothur and QIIME. The RDP Classifier is based on repeated random sampling (i.e., bootstrapping) of the k-mers belonging to a query sequence, and matching these k-mers to those from sequences in the training set [10]. Rather than using a measure of sequence divergence, confidence is calculated as the fraction of bootstrap replicates that were assigned to a given label (e.g., genus). Variations on this method have been proposed that claim to give higher accuracy, for example SINTAX and SPINGO [11, 12].

Machine learning classifiers often fail in situations where the correct label lies outside the scope of the training data [13]. For example, it has been demonstrated that the RDP Classifier has a relatively low misclassification rate on sequences that belong to groups in the training set [10, 14], but a much higher over classification rate on sequences belonging to novel groups that are unrepresented in the training set [11]. Over classifications are particularly detrimental in microbiome studies because many microorganisms are not represented in reference taxonomies [2, 15]. Two main approaches are currently employed to reduce over classifications: use of environment-specific training sets that decrease the number of unrepresented taxonomic groups [15, 16] and setting prior probabilities that lower the likelihood of assignment to an unexpected taxonomic group [17]. Both of these approaches require considerable prior knowledge about what microorganisms are expected in a sampled environment and, therefore, a more general solution to the problem of high over classification rates would be extremely useful.

Here, we introduce IDTAXA, a novel approach to taxonomic classification that shares features from phylogenetic, machine learning, and distance-based approaches. IDTAXA is able to lower over classification rates substantially across a variety of standard reference training sets. We compare IDTAXA to published classifiers that report a confidence for taxonomic assignment and scale well to large datasets. Impressively, IDTAXA achieves lower error rates than other methods while classifying the same fraction of classifiable sequences. Furthermore, we introduce novel algorithmic features that improve the practical utility of IDTAXA for classifying microbiome datasets, which may vary widely in the length and quality of their sequences. Finally, we show the implications of these attributes for the interpretation of human and environmental microbiome sequence data.

## Implementation

As with many other classifiers, the IDTAXA algorithm is split into two discrete phases: learning from a training set with the LearnTaxa function and classifying new query sequences with the IdTaxa function. The learning process only needs to occur once for each training set, resulting in a trained classifier that can be repeatedly used to classify as many sequences as desired with the IdTaxa function. Both functions are part of the R [18] package DECIPHER [19], which is distributed under the GPLv3 license as part of Bioconductor [20]. The LearnTaxa and IdTaxa functions are written in a combination of the C and R programming languages.

### The learning phase of the IDTAXA algorithm

The purpose of the LearnTaxa function is to identify putative problem sequences and problem groups in the training set and speedup the process of classifying new (query) sequences with the IdTaxa function. LearnTaxa takes a set of reference sequences and their respective taxonomic assignments (e.g., “Root; Bacteria; Proteobacteria; Gammaproteobacteria; Enterobacteriales; Enterobacteriacea; Escherichia”) as input. Consistent with standard definitions, the reference taxonomy is defined by a semicolon separated list of taxonomic names beginning with “Root;”, which collectively denote a multifurcating taxonomic tree. The root rank is defined as a catch-all for assigning sequences that do not fit into any lower taxonomic group, such as randomly generated sequences of A, C, G, and T. The reference taxonomy may contain as many rank levels as desired per group, for example the standard seven ranks (i.e., root, domain, phylum, class, order, family, and genus) or only a single rank level under the root rank. Optionally, rank level information (e.g., “genus” or “phylum”) for each group can be provided in “taxid” table format, which has been popularized by the RDP Classifier [6].

The LearnTaxa function decomposes each sequence into a set of overlapping, unique, and unambiguous (i.e., A, C, G, or T/U only) k-mers (i.e., subsequences of length *k*). By default, the value of *k* is chosen such that random k-mer matches between two sequences are expected roughly 1% of the time. For example, a training set containing full-length 16S rRNA gene sequences (~ 1500 nucleotides) would use a value of *k* = 8. Next, LearnTaxa records the top 10% of k-mers that best distinguish among the subgroups at each rank level, which we term the “decision k-mers.” For example, in the case of a 16S rRNA gene training set, at the root rank, it would record ~ 6500 k-mers that collectively indicate whether a sequence belongs to the Bacteria or Archaea. The criterion for determining the top decision k-mers at each rank level is based on the cross-entropy between a subgroup and its parent group [21]:

$$ \mathrm{cross}-{\mathrm{entropy}}_{ij}=-{p}_{ij}\times \log \left({q}_i\right) $$

where *p*_*ij*_ is the frequency of k-mer *i* relative to other k-mers in subgroup *j* and *q*_*i*_ is the frequency of k-mer *i* relative to other k-mers in its parent group. Therefore, the cross-entropy is maximized for k-mers that are frequent in their subgroup but rare in other subgroups, providing a set of k-mers that distinguish among subgroups optimally at each node of the taxonomic tree.

Finally, the LearnTaxa function attempts to reclassify each training sequence to its labeled taxonomic group using a method that we term “tree descent,” which is analogous to a decision tree commonly employed in machine learning algorithms (Additional file 1: Figure S1). Beginning at the top (i.e., Root) of the taxonomic tree, LearnTaxa samples a *fraction* (by default 6%) of the decision k-mers at each node (taxon) on the tree and removes k-mers that are not found in the query sequence. The group with the highest remaining sum of *p*_*ij*_ is recorded, and this process is repeated for 100 random bootstrap replicates (i.e., samples with replacement) of the decision k-mers. If a subgroup is selected in at least 80 bootstrap replicates, then the sequence descends the tree to this subgroup’s node, unless the subgroup is a terminal taxon. If the selected subgroup is incorrect for the reference sequence, or all subgroups are selected less than 80 (of 100) times, then the process terminates at the node.

During tree descent, the algorithm learns the optimal sampling *fraction* for each node on the taxonomic tree. If this *fraction* is too high (e.g., choosing all decision k-mers every bootstrap replicate), then the choice among subgroups is deterministic and prone to failure. If the *fraction* is too low (e.g., choosing one decision k-mer per bootstrap replicate), then the choice is too stochastic and does not adequately indicate which subgroup is most likely. Therefore, the *fraction* is initialized at a moderate value (by default 6%) at each node and is lowered when a reference sequence is assigned to an incorrect subgroup at a node. This process is repeated until (i) all sequences in the training set are correctly reclassified to their respective taxonomic group using tree descent, (ii) *fraction* decreases below a minimum value (by default 1%) at a specific node, or (iii) a maximum number of re-classification attempts (by default 10) are made for a sequence. Note that the value of *fraction* at a node is decreased with each failed attempt, which allows the classification at that node to improve in subsequent iterations.

Situation (ii) can occur when many reference sequences are assigned to the wrong subgroup at a specific node. Such taxonomic groups are recorded as putative “problem groups” and reported to the user. Situation (iii) can occur when the tree descent algorithm is confident that a reference sequence belongs in a certain subgroup, but this differs from its assigned taxonomy. The LearnTaxa function records these as putative “problem sequences” that are reported to the user. In practice, almost all reference sequences are correctly reclassified using tree descent, and the few reported problem sequences and problem groups correctly point to potential errors in the taxonomy (e.g., mislabeled sequences, groups placed into an incorrect subtree, or taxonomic groups that are not monophyletic). Ultimately, the tree descent process both serves the purpose of identifying errors in the taxonomy and speeding up the classification of query sequences with the IdTaxa function, as described next.

### The classification phase of the IDTAXA algorithm

The purpose of the IdTaxa function is to classify new (query) sequences as accurately and efficiently as possible. IdTaxa takes as input the object returned by the LearnTaxa function and a set of query sequences to classify. It returns a classification for each sequence in the form of a taxonomic assignment with associated confidences for each rank level (e.g., “Root [99%]; Bacteria [98%]; Proteobacteria [93%]; Gammaproteobacteria [89%]; Enterobacteriales [82%]; Enterobacteriaceae [80%]; Escherichia [32%]”). The classification is left unassigned below a user-specified confidence, by default 60%. For example, the above classification would end at “unclassified Enterobacteriaceae” because the genus level classification (*Escherichia*) falls below the default threshold of 60%. In this case, we could be reasonably confident that the microorganism belongs to the Enterobacteriaceae family, but we do not know the genus to which it belongs.

The IdTaxa function begins by splitting the query sequences into overlapping, unique, and unambiguous k-mers. Next, the tree descent process is commenced using the same strategy described for LearnTaxa, but requiring 98 (rather than 80) of 100 bootstrap replicates to continue descending the tree. The set of candidate taxa are determined according to the node where tree descent terminated, and the subset of reference sequences that are assigned to this taxon are used in subsequent stages (Additional file 1: Figure S1). In this way, IdTaxa only needs to consider classifying to a portion of the taxonomic tree, greatly accelerating the classification process for many query sequences.

The IdTaxa function now switches to subsampling k-mers of the query sequence rather than the decision k-mers. By default, IdTaxa samples *S* = *l*^0.47^ k-mers in each bootstrap replicate, where *l* is the length of the query sequence. If at most *S* unique k-mers exist in the sequence, then it is automatically assigned to unclassified Root at 0% confidence. We employ a text mining approach to weigh k-mer matches based on their inverse document frequency (IDF) [22, 23]. A k-mer’s *weight* is defined by the equation:

$$ {weight}_i=\log \left(n/\left(1+{f}_i\right)\right) $$

where *n* is the number of different taxa in the training set and *f*_*i*_ is the sum of the frequency of k-mer *i* across taxa. In this manner, the *weight* of very frequent k-mers approaches zero and the *weight* of very infrequent k-mers approaches log(*n*). The use of different weights for each k-mer is analogous to how different sites (i.e., columns) of an alignment can provide a variable amount of information when constructing a phylogenetic tree.

Unlike other algorithms, IDTAXA only selects a single representative sequence from each group in the training set to use for bootstrapping. This representative is chosen to be the sequence with the greatest total weight of k-mers from each terminal taxon. Selecting one sequence per group helps to correct for imbalance in the training set, where some groups have far more representatives than many other groups. For each bootstrap replicate, a sum of weights is calculated for the sampled k-mers that are found in each representative sequence, and the group with the highest total weight is selected as the “hit.” If multiple groups are tied for the maximum weight, as is the case when classifying a conserved sequence shared across several groups, then a random *hit* is selected.

The IdTaxa function then computes a confidence from the total weight of each group across bootstrap replicates. Unlike other classification methods that assign a confidence based on the number of bootstrap *hits*, the confidence reported by IdTaxa is also based on the *weight* of those *hits*. This modification makes the reported confidence better reflect the similarity between the query and its top *hit* in the training set. The formula used to calculate confidence is:

$$ {\mathrm{confidence}}_j=\sum \limits_{i=1}^B\left({d}_i/{d}_{\mathrm{avg}}\right)\times \left({h}_{ij}/{d}_i\right)=\sum \limits_{i=1}^B{h}_{ij}/{d}_{\mathrm{avg}} $$

where *h*_*ij*_ is the summed *weight* of all k-mers found in group *j* in bootstrap replicate *i*, *d*_*i*_ is the maximum possible summed *weight* in bootstrap replicate *i*, and *d*_*avg*_ is the average of *d*_*i*_ across all bootstrap replicates (*B*, by default 100). In other words, confidence is the fraction of the total possible *weight* assigned to a given group, which incorporates both the number of bootstrap replicates where it was the *hit* and how well it matched (i.e., its k-mer distance). In this way, it is possible for a group to be the *hit* in all bootstrap replicates but still have a low confidence. Finally, the highest confidence basal group (e.g., genus) is selected, and confidences are recursively summed to higher rank levels up the tree.

### Programs used for benchmark comparisons

The IDTAXA algorithm is implemented in the R [18] package DECIPHER [19] version 2.6.0. We focused on benchmarking against the RDP Classifier (v2.12) because it is widely used and has repeatedly been demonstrated to be one of the best classification methods [6]. We also compared against more recent programs that have been shown to outperform the RDP Classifier: MAPSeq (v1.2.2) [24, 25], QIIME 2 q2-feature-classifier (v2018.6.0) [17], SPINGO (v1.3) [12], and SINTAX (v9.2.64) [11]. We omitted other classification programs because they generated errors during benchmarking, were too slow to run leave-one-out cross-validation, or were unpublished. As a representative of nearest neighbor methods, we included local and global percent identity as determined from the top BLAST (v2.6.0) [26] hit with the excluded sequence as the query and the remaining training set as the subject.

In some cases, we report classification results at a program-specific confidence: BLAST (95% identity), QIIME (70% confidence), IDTAXA (60% confidence), MAPSeq (50% confidence), and SINTAX, SPINGO, and the RDP Classifier (80% confidence). These thresholds were selected because they are the programs’ default/recommendation or are commonly used for full-length 16S rRNA gene sequences. We selected a default value of 60% (very high confidence) for IDTAXA because it provided a conservative classification with relatively minimal MC and OC error rates. Less conservative thresholds, such as 50% (high confidence) or 40% (moderate confidence), could be specified if a user would prefer to have more sequences classified at the expense of higher error rates. Note that BLAST, QIIME, and SPINGO only provide a single confidence value, so this confidence was propagated to every rank level. For example, we considered a sequence with 90% confidence at the genus level to have 90% confidence at every level up to, and including, the root rank.

### Training sets used for classification benchmarking

Three reference datasets were used to evaluate the performance of different classifiers with leave-one-out cross-validation (Additional file 1: Figure S2). The most popular of these is the 16S training set (version 16) provided by the Ribosomal Database Project (RDP), consisting of 2472 genera [6]. The RDP training set is highly imbalanced, with 1119 (45%) singleton genera having only one sequence representative and, at the other extreme, a single genus (*Streptomyces*) having 594 sequences. We also extracted the V4 region (*Escherichia coli* positions 534–786) of the 16S rRNA gene from these sequences to create a test set that reflected the shorter lengths of reads obtained from current sequencing technologies. As an alternative to the RDP training set, we used the contax.trim (Contax) training set, which contains 38,781 full-length 16S rRNA gene sequences [27]. The Contax training set consist of 1774 genera that have a consensus taxonomy shared across multiple sequence repositories, of which only 156 are singleton genera.

To investigate the broader applicability of each classifier to other types of sequences, we compared performance on the Warcup (version 2) Fungal ITS training set [28]. The internal transcribed spacer (ITS) is the region between the small and large subunits of the ribosomal RNA operon. The Warcup dataset was constructed by clustering sequences at high similarity (> 97% identity), manually correcting inconsistencies in labeling, and then reclassifying the training sequences with the RDP Classifier using the training sequences themselves as the training set. It contains 17,878 sequences assigned to 8551 species, of which 2262 are singleton species. Note that both the 16S training set and Warcup use a taxonomy with a varying number of rank levels. A standardized taxonomy was used as input for MAPSeq and SINTAX since both classifiers require a fixed set of rank levels.

### Determining accuracy with leave-one-out cross-validation

To compare classifiers, leave-one-out cross-validation was performed by removing one sequence at a time, retraining the classifier with the remainder of the training set, and reclassifying the excluded sequence. For each excluded sequence, we recorded its predicted taxonomic classification and confidence at each rank level. This presents two possible types of error depending on whether the excluded sequence was the only representative of its group in the training set (i.e., a singleton) or other sequence representatives from this group remained in the training set. Misclassification errors occur when a sequence is incorrectly reclassified at a confidence ≥ threshold, and the correct group was present in the training set even after leaving out the sequence. Over classification errors occur when a sequence is assigned to any group at a confidence ≥ threshold, and the correct group did not exist in the training set after leaving out the sequence (i.e., a singleton).

Importantly, confidences cannot be directly compared across programs because a given confidence (e.g., 90%) may not have equivalent meaning. Therefore, we recorded the fraction of classifiable sequences that are classified, also known as 1—the under-classification rate [29], at each confidence level and compared misclassification (MC) and over classification (OC) error rates at the same fraction of classifiable sequences classified. Classifiable sequences are defined as those whose group remains even after exclusion from the training set, that is, those that have the potential to cause an MC error. Therefore, the fraction of classifiable sequences classified is the fraction of non-singleton sequences in the training set that were classified above a given confidence threshold during leave-one-out cross-validation. To have greater accuracy, a program must have lower MC and/or OC error rates while classifying the same fraction of classifiable sequences. Notably, this result is independent of the relative scaling of confidence values across programs, and any monotonic transformation (e.g., square root) of reported confidences would yield the same result. Furthermore, we weighed the sequences from each basal taxon (e.g., genus) equally when calculating the MC error rate to prevent extremely over-represented groups (e.g., *Streptomyces* in the RDP training set) from dominating the error rate during leave-one-out cross-validation.

Note that we report the fraction of classifiable sequences classified rather than the fraction of total sequences classified. This is preferable because it prevents us from penalizing when classifiers leave unclassifiable sequences unclassified. For example, consider the case where the OC error rate is lowered but the MC error rate is held constant. This would result in fewer total sequences classified at a given confidence, which would make a classifier appear both better (i.e., lower OC error rate) and worse (i.e., fewer total sequences classified) in different respects. However, the fraction of classifiable sequences classified would remain unchanged when the MC error rate is held constant, and decreasing the OC error rate would rightly appear as an improvement. This adequately reflects the goal of classification, which is to correctly assign as many sequences as possible while withhold assignment of sequences belonging to groups that are unrepresented in the training set.

## Results

### The IDTAXA algorithm exhibits lower over classification error rates

**Fig. 1:**
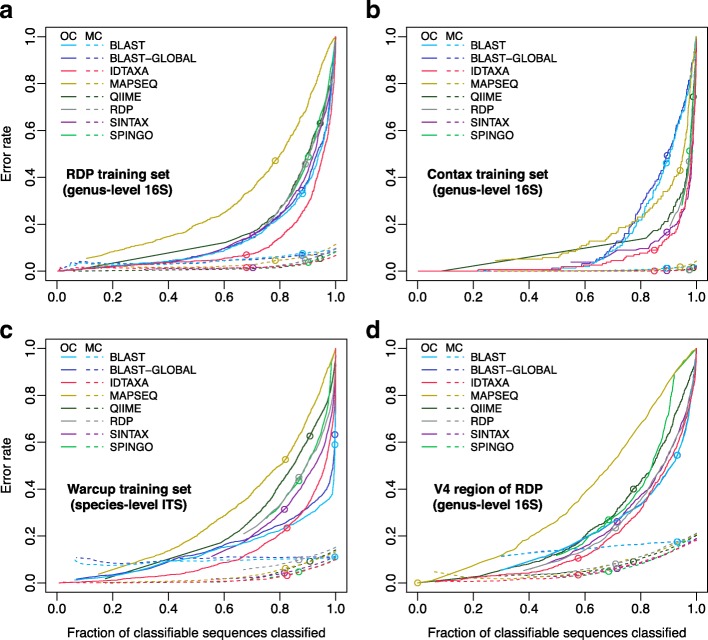
The IDTAXA algorithm exhibits relatively low OC error rates. Plots showing error rates versus the fraction of classifiable sequences classified as confidence is varied from 100% (left) to 0% (right). A better classifier will exhibit lower error rates during leave-one-out cross-validation while classifying the same fraction of classifiable sequences, shifting its curves downward. Misclassification (MC) error rates (dashed lines) are much lower than over classification (OC) error rates (solid lines) on three different training sets: the RDP training set of full-length 16S rRNA gene sequences (**a**), the Contax training set (**b**), and the Warcup ITS training set (**c**). The IDTAXA algorithm consistently displays the lowest OC error rates across different training sets. MC and OC error rates are higher when testing the shorter V4 region (~ 251 nucleotides) of the RDP training set (**d**). Points indicate error rates at default/recommended confidence thresholds: ≥ 95% sequence identity for BLAST, ≥ 70% confidence for QIIME, ≥ 60% confidence for IDTAXA, ≥ 50% confidence for MAPSeq, and ≥ 80% confidence for all others

We focused on the basal taxonomic rank (e.g., genus or species) in each training set for benchmarking classification accuracy because the basal rank is the most difficult to predict. Setting the confidence threshold to zero provides a classification for all sequences, which results in an over classification (OC) error rate of 100% and a maximal misclassification (MC) error rate. At the other end of the spectrum, setting the confidence threshold to 100% minimizes error rates but classifies the smallest fraction of sequences. Figure 1 shows the MC and OC error rates for different classifiers on the popular RDP training set for 16S rRNA gene sequences. Better classifiers yield lower error rates while classifying the same fraction of classifiable sequences, resulting in curves that are further toward the bottom-right corner of the plot.

It is apparent from Fig. 1a that IDTAXA has a substantially lower OC error rate than the other classifiers across the entire range of confidence thresholds on the RDP training set. The nearest neighbor (BLAST) approach provides lower OC error rates than the other methods but higher MC error rates. The QIIME and SPINGO algorithms yielded lower MC error rates than the RDP Classifier, but similar OC error rates. The SINTAX algorithm is nearly identical to the RDP Classifier in MC error rate, but has slightly lower OC error rates. SINTAX is described as having a substantially lower error rate than the RDP Classifier [11], but this appears to be due primarily to SINTAX classifying a lower fraction of classifiable sequences at the same confidence threshold as the RDP Classifier (i.e., 80%). Notably, we observe the same pattern for all rank levels, although error rates decrease at higher ranks as expected (Additional file 1: Figure S3).

To determine whether IDTAXA’s improved performance was independent of the training data, we compared our results across multiple training sets. Benchmarking on the Contax training set generally resulted in lower error rates (Fig. 1b), suggesting that this training set may harbor fewer labeling errors than the RDP training set. The classifiers’ performance ranking was similar with the exception of BLAST, which performed far more poorly on Contax than the RDP training set. Next, we compared the classifiers on the Warcup (ITS) training set, which yielded a similar result to the RDP training set (Fig. 1c). The biggest difference from the RDP training set was for the RDP Classifier, which had much higher MC error rates. Notably, BLAST’s curve for OC error rate appears to have a kink, which may be related to the fact that the Warcup training set was partly constructed using BLAST [28]. Taken together, these results confirmed the high accuracy of the IDTAXA algorithm for taxonomic classification across multiple training sets.

Leave-one-out cross-validation has been criticized because sequences may remain in the training set that are closely related to the query sequence. Recently, cross-validation by identity has been proposed as a viable alternative, whereby the entire training set and test set do not contain any sequences within a specified percent similarity [29]. We used the TAXXI benchmark to test whether IDTAXA offers superior accuracy to other classifiers at its lowest rank level (species) and a corresponding similarity cutoff (≤ 97%) that would ensure all closely related sequences were absent from the training set. On both the BLAST 16S and Warcup ITS benchmarks, IDTAXA outperformed all other classifiers, with lower MC and OC error rates across all under-classification rates (Additional file 1: Figure S4). Therefore, the independent TAXXI benchmark confirmed IDTAXA’s superior ability to accurately classify microbiome sequences.

**Fig. 2:**
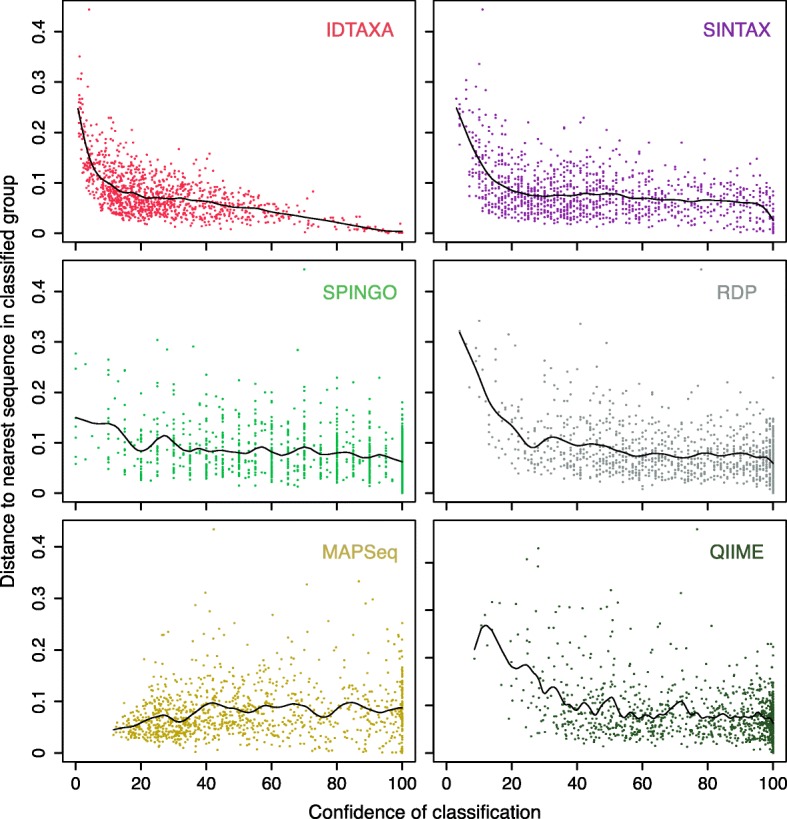
Variability in sequence similarity at the same confidence level. During leave-one-out cross-validation with the RDP training set, for each singleton sequence, we computed the distance to the nearest sequence in the group to which it was assigned. The IDTAXA algorithm only assigned a high confidence to sequences that had a low distance to the query sequence being classified. In contrast, all other k-mer approaches assigned high confidences even when all of the sequences in the group were distant to the query sequence. The curves indicate the cubic spline that best fits the data

We wished to better understand why the IDTAXA algorithm outperforms other classification algorithms. Figure 2 shows that, for singleton sequences, IDTAXA assigns confidences that are better correlated with the distance between the sequence and the nearest sequence in its assigned group. In particular, all other approaches assigned some query sequences high confidence even though they are greater than 10% distant from the assigned sequence. Since IDTAXA combines both k-mer distance and bootstrapping into its confidence measure, it is able to avoid assigning a high confidence to sequences even if they repeatedly are selected as the top *hit* during bootstrapping. Moreover, unlike other algorithms, IDTAXA down-weights conserved k-mers that provide minimal power to resolve taxonomic groups.

### IDTAXA maintains low error rates across varying input sequence lengths

Having confirmed that the IDTAXA algorithm is accurate on a training set of mostly full-length sequences, we sought to understand performance on shorter sequences that are common in microbiome sequence datasets. We noted that the degree of stochasticity introduced during bootstrapping is based on the relative number of samples (*S*) drawn from the total set of *l* k-mers belonging to a sequence. The RDP Classifier draws one eighth of the k-mers (*S* = *l*/8) in a sequence for each bootstrap replicate, whereas the SINTAX algorithm always draws 32 k-mers independently of query sequence length (*S* = 32). Rather than arbitrarily choosing a function *S*(*l*) for drawing k-mers during bootstrapping, we examined this function using subsequences of a simulated training set of 1000 sequences with 90,000 nucleotides each [30]. Full-length sequences were clustered at ≥ 95% similarity, resulting in 607 groups.

Using this taxonomy as the training set, we calculated OC error rates for varying bootstrap sample sizes (*S*) as a function of subsequence length (*l* = 32 to 8192). When the OC error rate is held constant, we observe that *S*(*l*) follows an apparent power-law scaling with *S*(*l*) = *l*^*x*^, where *x* is a positive constant greater than zero and less than 1 (Additional file 1: Figure S5). We chose the fixed point of 10% OC error rate at 1600 nucleotides to define *x* as 0.47. While other values of *x* could be chosen, 0.47 was selected because it results in sampling most of the k-mers belonging to sequences of typical length (250–2000 nucleotides) across at least one of the 100 bootstrap replicates. Notably, *x* has negligible bearing over the MC and OC error curves in Fig. 1, although it does change where the confidence threshold (e.g., 60%) is situated on the curve.

Even though the OC error rate is largely independent of query sequence length, the MC error rate decreases for longer sequences (Additional file 1: Figure S6). Similarly, the fraction of classifiable sequences that are classified continues to improve with longer sequences. Thus, it is preferable to use the longest sequences possible for classification even though the OC error rate will probably not change significantly. While we expect this behavior to stay consistent across sequence types (e.g., 16S or ITS), the actual error rates are dependent on the training set and cannot be inferred from the simulated sequences. Therefore, we did not compare the performance of the IDTAXA algorithm to any other classifiers using the simulated training set. Nevertheless, it is worth noting that the IdTaxa function allows users to specify other forms of *S*(*l*) as desired (e.g., *S*(*l*) = 32 or *S*(*l*) = *l*/8).

We wished to know how the input sequence length affected the accuracy of different algorithms on a real training set. To benchmark shorter length sequences, we performed leave-one-out cross-validation on the RDP training set while testing a ~ 251 bp subsequence corresponding to the V4 region of the 16S rRNA gene extracted from the full-length RDP training set. This variable region is frequently selected for sequencing and, thus, represents a common test case for classifying short sequences. As expected, the accuracy of all algorithms diminished for shorter sequences, although the IDTAXA algorithm continued to display lower OC error rates than other programs (Fig. 1d). Importantly, the OC error rate remained approximately the same on full-length and shorter test sequences for IDTAXA, even though the fraction of sequences classified decreased for the same confidence threshold (60%). In contrast, OC error rates changed considerably for all other programs at their respective default thresholds (Fig. 1a, d). This provides a practical advantage for IDTAXA users because a single threshold can be used for input sequences of different lengths with the reassurance that the primary mode of classification error (OC errors) will not increase dramatically for some sequences over others. In comparison, the RDP Classifier documentation suggests adjusting the confidence threshold to 50% for sequences shorter than 250 bp [31].

### Performance on random and repeat sequences

**Fig. 3:**
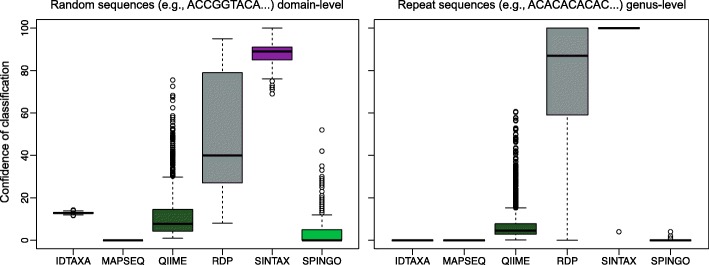
Confidences assigned to random and repeat sequences. Using the RDP training set, the RDP Classifier and SINTAX assigned high confidences at the domain level (i.e., Bacteria or Archaea) to 1000 query sequences composed of 1000 random nucleotides. Similarly, both the RDP Classifier and SINTAX assigned high confidence at the genus level to 1000 sequences composed of repeats with periodicity varying from 1 (e.g., AAA...) to 7. In contrast, the IDTAXA, MAPSeq, and SPINGO algorithms assigned low confidences to random and repeat sequences at all taxonomic levels

It has been anecdotally reported that some programs return high confidence classifications for randomly generated sequences and sequences composed solely of repeats (e.g., ACACAC...). To investigate this phenomenon, we generated 1000 random sequences with a 25% probability of each nucleotide and 1000 sequences with repeat periodicity varying from 1 (e.g., AAA...) to 7. All sequences were of length 1000 to reflect typical sequence lengths used for classification. Figure 3 shows that the RDP Classifier and SINTAX often assign high confidence to random sequences at the domain level when using the RDP training set. In contrast, all other classifiers, including IDTAXA, assign relatively low confidence to random sequences. Furthermore, the RDP Classifier and SINTAX often assign high (80–100%) confidence at the genus level to repeat sequences. This is because a small number of sequences in the training data sometimes contain one or more of the unique k-mers that comprise a repeat sequence. This results in a single taxonomic group appearing as the top *hit* in nearly every bootstrap replicate. IDTAXA effectively avoids this problem by assigning 0% confidence to sequences having at most *S*(*l*) unique k-mers, for which bootstrapping (i.e., sampling with replacement) would result in a high number of repeated k-mers per bootstrap replicate.

### Mock community sequences recapitulate the benchmarking results

Having demonstrated the merits of the IDTAXA algorithm through leave-one-out cross-validation, we compared the ability of classification programs to detect the organisms present in a mock microbial community. We focused on a mock microbiome (Microbial Community C) provided by the Human Microbiome Project [32] that had previously been Illumina sequenced (accession SRR3225706) as part of a different study [33]. This mock community is composed of strains belonging to 20 different bacterial genera, all of which are represented in the RDP training set. The dataset set contains 14,072 sequences (median length 374 nucleotides) amplified with V4-V5 primers after extraction with the QIAamp kit.

Results of classifying with each of the different classification programs are summarized in Table 1. All classifiers assigned between 93 and 98% of sequences to the genus rank at their default/recommended confidence thresholds. The BLAST and SPINGO algorithm both identified 17 of the 20 expected genera, QIIME identified 16, the RDP Classifier and MAPSeq identified 15, and both SINTAX and IDTAXA identified 14. However, BLAST also identified 24 unexpected genera that were not present in the sample, the RDP Classifier identified 7, MAPSeq and QIIME identified 6, and SPINGO and SINTAX identified 3. IDTAXA only identified 2 unexpected genera, *Prevotella* and *Aquabacterium*, both of which were also present in almost all other programs’ classifications. It also identified one unexpected family, *Comamonadaceae*, that includes the genus *Aquabacterium*. Interestingly, the sequences corresponding to these unexpected groups were distant from any of the known 16S rRNA gene sequences included in the mock microbiome sample, suggesting that they were likely artifacts of contamination [34, 35].

**Table 1:** Number of taxonomic groups identified by each classifier among Illumina 16S rRNA gene sequences (SRR3225706) from a mock microbiome sample [33]. Counts are provided with and without including any sequences in the RDP training set that are labeled as belonging to the 20 expected genera

		Classified to genus level^α^ (%)	Groups present in the mock community							Absent from mock community^β^		
			Root	Domain	Phylum	Class	Order	Family	Genus	Order	Family	Genus
Using the RDP training set	BLAST	97.9	1	0	0	0	0	0	17	0	0	24
	IDTAXA	94.2	1	0	1	1	2	5	14	0	1	2
	MAPSeq	96.5	1	0	0	0	0	4	15	0	2	6
	QIIME	95.4	1	0	0	0	0	0	16	0	0	7
	RDP Classifier	93.3	1	1	2	3	6	8	15	0	2	6
	SINTAX	94.2	1	1	1	4	3	3	14	1	0	3
	SPINGO	96.5	1	0	0	0	0	0	17	0	0	3
With expected genera excluded from training data	BLAST	17.3	1	0	0	0	0	0	0	0	0	65
	IDTAXA	0.01	1	1	1	2	3	4	0	0	2	2
	MAPSeq	24.6	1	0	0	2	5	11	0	1	8	20
	QIIME	13.5	1	0	0	0	0	0	0	0	0	16
	RDP Classifier	3.83	1	1	2	3	6	9	0	0	3	12
	SINTAX	8.76	1	1	1	7	5	6	0	1	1	9
	SPINGO	26.7	1	0	0	0	0	0	0	0	0	15

^α^Percent of total sequences from the mock community that were classified to the genus rank

^β^Other rank levels (root, domain, phylum, and class) all had counts of zero

Since all of the expected genera were already present in the RDP training set, the above approach could only confirm the relatively high MC error rates of some classifiers. To investigate OC error rates, we removed the sequences corresponding to the 20 expected genera from the RDP training set and reclassified the mock community sequences. The results (Table 1) further confirmed that all programs other than IDTAXA suffer from considerable over classifications when the correct group is not present in the training data. IDTAXA only added a single unexpected family, *Planococcaceae*, while all other classification programs substantially increased their number of over classifications at the genus rank to between 9 and 65. Impressively, without the expected groups present in the training set, IDTAXA only classified 0.01% of sequences to the genus rank, in sharp contrast to the 3.8–26.7% of sequences classified to the genus rank by the other classification programs. Taken together, these results demonstrate that IDTAXA’s comparably low MC and OC error rates on benchmarks also extend to mock community microbiome sequences.

### IDTAXA’s classifications change the interpretation of microbiome data

We next sought to determine whether IDTAXA’s improved accuracy had a substantial effect on the interpretation of human and environmental microbiome samples. We decided to focus on comparing to the RDP Classifier because it is currently the most popular classification approach. To this end, we selected full-length 16S rRNA gene sequences collected from the human gut of an adult male and a compilation of different sediment samples with high bacterial and archaeal diversity [2]. The number of reads assigned to each group in the RDP training set was compared at the default confidence threshold recommended for IDTAXA (60%) and the RDP Classifier (80%). Since the RDP Classifier is more permissive than IDTAXA, we repeated the analysis using a maximal (100%) confidence threshold with the RDP Classifier.

**Fig. 4:**
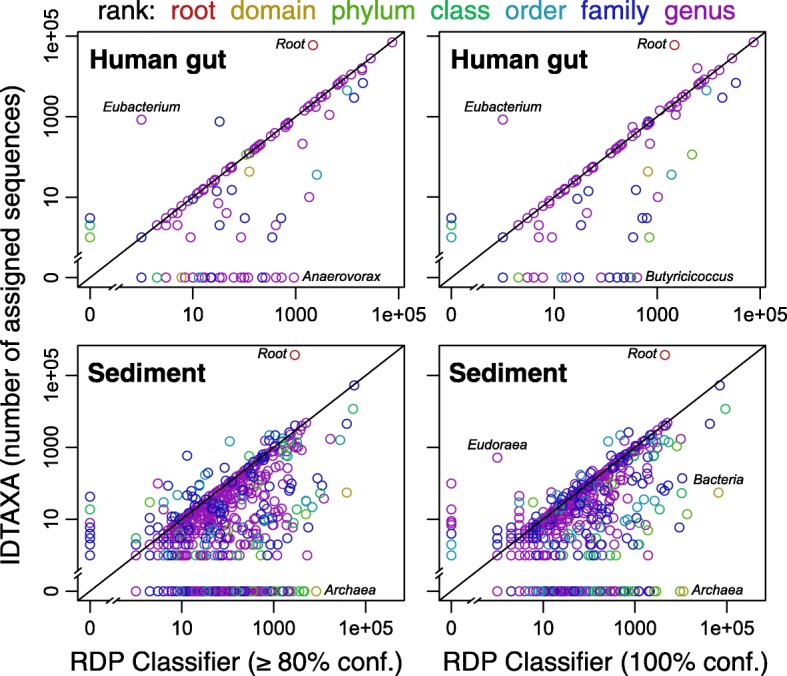
Comparison of classifications using human and environmental microbiome data. The number of sequences assigned to each taxonomic group in the RDP training set is shown for full-length 16S rRNA gene sequences originating from two different environments [2]. The RDP Classifier was far more permissive at its default (≥ 80%) confidence than IDTAXA at its default (≥ 60%) confidence. Even at a 100% confidence threshold, the RDP Classifier assigned sequences to many more groups than the IDTAXA algorithm, possibly because of its substantially higher OC error rate. Note that some points may be overlapping, particularly at low numbers of assigned sequences

Figure 4 illustrates the four major conclusions of this comparison on human and environmental microbiome data. First, both the RDP Classifier and IDTAXA agree on the presence of many groups, and often assign a similar number of reads to the same groups. Second, the IDTAXA algorithm tends to leave sequences unclassified at the root rank rather than classifying them to either Bacteria or Archaea, as seems to be the preference of the RDP Classifier. Third, there are an extremely high number of groups assigned by the RDP Classifier that the IDTAXA algorithm does not indicate are present. Even with a 100% confidence threshold, the RDP Classifier assigned sequences to 12 genera in the human gut and 138 genera in the sediment sequences that IDTAXA did not find present. In sharp contrast, IDTAXA classified zero genera in human gut sequences and only 22 genera in sediment sequences that the RDP Classifier did not identify. Forth, IDTAXA assigned fewer sequences to low rank levels (e.g., genus) than the RDP Classifier, as we had observed with the mock community analysis. IDTAXA classified 5.3% of sequences from sediment to the genus level and 19.9% of sequences from the human gut. In contrast, RDP classified 17.7% (≥ 80% confidence) and 9.5% (100% confidence) of the sediment sequences, as well as 22.5% and 20.0% of the human gut sequences, respectively.

Since these classifications were performed on human and environmental microbiome samples, we do not know the true community of microorganisms that were present. However, based on the aforementioned analyses, it is likely that most of the taxonomic groups that are unique to the RDP Classifier are false positive classifications caused by the lack of the correct taxonomic group in the training data. We also noted that many of these unique groups had relatively high abundance. By comparison, groups that were uniquely assigned by IDTAXA tended to have relatively low read counts (Fig. 4). High abundance over classifications could easily lead to incorrectly interpreting the known diversity in microbiome studies, as well as leading to incorrect conclusions about the groups that are part of a microbiome. Furthermore, based on the mock community analysis, it is likely that the RDP Classifier is classifying sequences to lower rank levels (e.g., genus) than feasible, resulting in incorrect classifications.

### IDTAXA exhibits sub-linear scalability with reference training set size

As with other classifiers [17], DECIPHER scales linearly in time with the number of unique query sequences because input sequences are processed independently. To evaluate performance, we measured runtimes on the largest training set (Contax) with increasing numbers (*N*) of reference sequences (Additional file 1: Figure S6) while maintaining the number of query sequences at 1000. SINTAX was generally the fastest method tested, except at the highest number of training sequence (*N* = 35,000) where RDP was the fastest. BLAST was the slowest method, requiring seconds to process each query sequence, and making it impractical to use on large sequence sets. IDTAXA was about 10-fold slower than SINTAX, requiring 0.05 to 0.3 s per query sequence depending on the size of the reference training set (*N*). This was expected given that IDTAXA needs to perform more computations than many other k-mer matching algorithms and there is a trade-off between speed and accuracy. Notably, we parallelized the step of the IDTAXA algorithm that requires comparison to reference sequences, allowing IDTAXA to achieve approximately fourfold speedup when using eight processors.

To evaluate scalability, we fit a power-law function (*T*~*aN*^*b*^) to the measured runtimes for each classifier (Additional file 1: Figure S7). Runtimes scaled roughly linearly for SINTAX (*T∝N*^1.05^) and greater than linearly for MAPSEQ (*T∝N*^1.61^). IDTAXA displayed sub-linear scalability when using one (*T∝N*^0.87^) or eight (*T∝N*^0.67^) processors, which is the result of speedups achieved during the tree descent phase of the algorithm that exploit hierarchical structure in the reference taxonomy. IDTAXA’s scalability was similar to that of SPINGO (*T∝N*^0.89^) and BLAST (*T∝N*^0.72^). The RDP Classifier (*T∝N*^0.13^) and QIIME (*T∝N*^0.09^) had the best scalability. In terms of maximum memory usage (*M*), IDTAXA exhibited sub-linear scalability (*M∝N*^0.5^), requiring a maximum of about 1.5 GB on the largest reference set tested (*N* = 35,000). IDTAXA’s primary usage of memory space is for storing decision k-mers used during the tree descent phase of the algorithm. The number of decision k-mers is proportional to the number of reference groups, which tends to scale sub-linearly with the number of reference sequences.

## Discussion

**Fig. 5:**
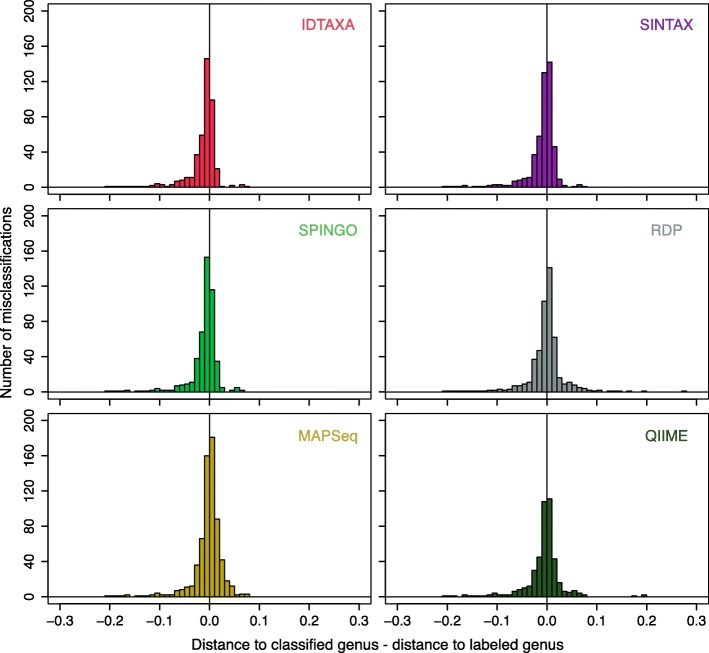
Some misclassifications may be due to labeling errors. Many misclassifications (≥ 0% confidence) on the full-length RDP training set are to groups containing a sequence that has greater sequence identity than any sequence in the correct group. Extreme cases to the left of the vertical line are potentially due to labeling errors in the RDP training set

Throughout this work, we made the assumption that the taxonomic assignments of training sequences were unequivocally correct. Yet, as demonstrated by the discrepancy in accuracy between the Contax and RDP training sets, it is highly likely that taxonomies contain errors. As further proof, we observed that MC errors were often much more similar to the group they were assigned than they were to the nearest sequence in their “correct” group (Fig. 5). However, we cannot rule out the fact that the distance between 16S rRNA gene sequences is only a proxy for taxonomic relatedness, and that taxonomic assignments are often based on many factors, such as the core genome, that may disagree with the 16S rRNA gene phylogeny. Furthermore, full-length 16S rRNA gene sequences do not always offer sufficient resolution to distinguish between taxonomic groups, as has repeatedly been shown to be the case for species-level taxonomic assignments [36–40].

These discrepancies raise the important question of which training set is best for classification. Training sets differ considerably in their number of sequences, scope, degree of imbalance, and accuracy of labels. IDTAXA provides a means of differentiating among training sets because it flags putative problem sequences and problem groups during its learning phase. We have noted that the RDP training set, which is one of the most popular, has many putative labeling errors according to LearnTaxa, whereas the Contax training set has fewer errors but narrower scope. We favor the GTDB [41], which is a relatively new training set based on a standardized taxonomy and has relatively few putative errors flagged by LearnTaxa. Since the GTDB taxonomy is based on genomes, its scope is likely to continue to expand in the future.

## Conclusions

Here, we have shown that IDTAXA substantially reduces false positive classifications of test sequences falling outside the scope of a training set. Over classifications are particularly problematic in microbiome research as only a fraction of existing microbial diversity is represented in even the largest training sets such as the SILVA database [2]. IDTAXA mitigates OC errors by taking a hybrid approach that combines features of phylogenetic, distance-based, and machine learning classification methods. This helps to circumvent the main weakness of purely machine learning approaches, which is that they are poor at identifying when test data belongs to a novel label. The hybrid approach employed here may be applicable to other classification problems in biology where the training dataset is incomplete.

**Fig. 6:**
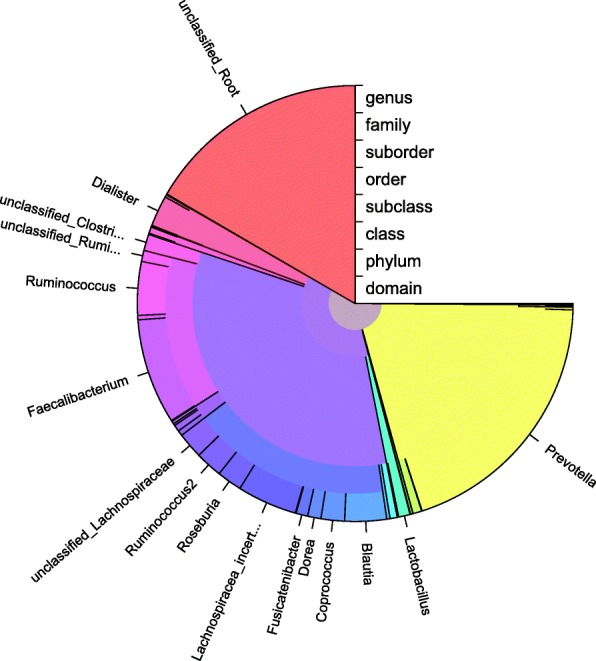
Result of classifying sequences with the IdTaxa function. The outputs of the IdTaxa function can be plotted with the DECIPHER package for the R programming language or exported for integration into a separate bioinformatics pipeline. The pie chart shows the distribution of IDTAXA classifications for 268,930 full-length 16S rRNA gene sequences from a human gut sample [2]

The IDTAXA algorithm has been implemented in the DECIPHER package for the R programming language and is available from Bioconductor. The documentation describes how to train the classifier on a new training set, which can be composed of any type of sequence (e.g., 16S, ITS, or other). A variety of pre-trained training sets are available from the website http://DECIPHER.codes/. We have also made available a webserver that will classify sequences using any of these training sets. The code and webserver are both able to generate plots (e.g., Fig. 6) that allow users to visualize their sequences’ classifications, and the classifications are exportable to standard tabular formats so that users can integrate the results into their own bioinformatics pipeline.

## Availability and requirements

Project name: DECIPHER

Project home page: http://DECIPHER.codes

Operating system(s): Platform independent

Programming language: R and C

Other requirements: R 3.3 and higher

License: GNU GPL

Any restrictions to use by non-academics: None

## Additional file

Additional file 1: Supplemental figures S1-S7. (PDF 764 kb)

## References

[1] Nussinov R, Papin JA (2017). How can computation advance microbiome research?. PLoS Comput Biol.

[2] Karst SM, Dueholm MS, McIlroy SJ, Kirkegaard RH, Nielsen PH, Albertsen M (2018). Retrieval of a million high-quality, full-length microbial 16S and 18S rRNA gene sequences without primer bias. Nat Biotech.

[3] Parks DH, Rinke C, Chuvochina M, Chaumeil P-A, Woodcroft BJ, Evans PN, et al. Recovery of nearly 8,000 metagenome-assembled genomes substantially expands the tree of life. Nat Microbiol. 2017;2:1533–42. 10.1038/s41564-017-0012-7 28894102

[4] Rinke C, Schwientek P, Sczyrba A, Ivanova NN, Anderson IJ, Cheng J-F (2013). Insights into the phylogeny and coding potential of microbial dark matter. Nature.

[5] Altschul SF, Madden TL, Schäffer AA, Zhang J, Zhang Z, Miller W (1997). Gapped BLAST and PSI-BLAST: a new generation of protein database search programs. Nucleic Acids Res. Oxford Univ Press.

[6] Wang Q, Garrity GM, Tiedje JM, Cole JR (2007). Naive Bayesian classifier for rapid assignment of rRNA sequences into the new bacterial taxonomy. Appl Environ Microbiol.

[7] Nguyen N-P, Mirarab S, Liu B, Pop M, Warnow T (2014). TIPP: taxonomic identification and phylogenetic profiling. Bioinformatics.

[8] Golob JL, Margolis E, Hoffman NG, Fredricks DN. Evaluating the accuracy of amplicon-based microbiome computational pipelines on simulated human gut microbial communities. BMC Bioinformatics. 2017;18:283. 10.1186/s12859-017-1690-0 PMC5450146 28558684

[9] Zheng Q, Bartow-McKenney C, Meisel JS, Grice EA (2018). HmmUFOtu: an HMM and phylogenetic placement based ultra-fast taxonomic assignment and OTU picking tool for microbiome amplicon sequencing studies. Genome Biol.

[10] Vinje H, Liland KH, Almøy T, Snipen L (2015). Comparing K-mer based methods for improved classification of 16S sequences. BMC Bioinformatics..

[11] Edgar R. SINTAX: a simple non-Bayesian taxonomy classifier for 16S and ITS sequences. bioRxiv; 2016;1:1–10.

[12] Allard G, Ryan FJ, Jeffery IB, Claesson MJ (2015). SPINGO: a rapid species-classifier for microbial amplicon sequences. BMC Bioinformatics.

[13] Dave RN (1991). Characterization and detection of noise in clustering. Pattern Recogn Lett.

[14] Liu KL, Porras-Alfaro A, Kuske CR, Eichorst SA, Xie G (2012). Accurate, rapid taxonomic classification of fungal large-subunit rRNA genes. Appl Environ Microbiol.

[15] Rohwer RR, Hamilton JJ, Newton RJ, McMahon KD. TaxAss: Leveraging Custom Freshwater Database Achieves Fine-Scale Taxonomic Resolution. bioRxiv. 2018;1:1–37. 10.1128/mSphere.00327-18 PMC6126143 30185512

[16] Choi J, Yang F, Stepanauskas R, Cardenas E, Garoutte A, Williams R, et al. Strategies to improve reference databases for soil microbiomes. The ISME Journal. 2017;11:829–34. 10.1038/ismej.2016.168 PMC5364351 27935589

[17] Bokulich NA, Kaehler BD, Rideout JR, Dillon M, Bolyen E, Knight R, et al. Optimizing taxonomic classification of marker-gene amplicon sequences with QIIME 2's q2-feature-classifier plugin. Microbiome. 2018;6:90. 10.1186/s40168-018-0470-z PMC5956843 29773078

[18] R Core Team (2018). R: a language and environment for statistical computing [Internet].

[19] Wright ES (2016). Using DECIPHER v2.0 to analyze big biological sequence data in R. R Journ.

[20] Gentleman RC, Carey VJ, Bates DM, Bolstad B, Dettling M, Dudoit S (2004). Bioconductor: open software development for computational biology and bioinformatics. Genome Biol.

[21] Goodfellow I, Bengio Y, Courville A (2016). Deep learning.

[22] Jones KS (1972). A statistical interpretation of term specificity and its application in retrieval. J Doc.

[23] Robertson S (2005). Understanding inverse document frequency: on theoretical arguments for IDF. J Doc.

[24] Matias Rodrigues JF, Schmidt TSB, Tackmann J, Mering von C (2017). MAPseq: highly efficient k-mer search with confidence estimates, for rRNA sequence analysis. Bioinformatics.

[25] Almeida A, Mitchell AL, Tarkowska A, Finn RD. Benchmarking taxonomic assignments based on 16S rRNA gene profiling of the microbiota from commonly sampled environments. Gigascience. 2018;7 10.1093/gigascience/giy054. 10.1093/gigascience/giy054 PMC5967554 29762668

[26] Altschul SF, Gish W, Miller W, Myers EW, Lipman DJ (1990). Basic local alignment search tool. J Mol Biol.

[27] Liland KH, Vinje H, Snipen L (2017). microclass: an R-package for 16S taxonomy classification. BMC Bioinformatics.

[28] Deshpande V, Wang Q, Greenfield P, Charleston M, Porras-Alfaro A, Kuske CR, et al. Fungal identification using a Bayesian classifier and the Warcup training set of internal transcribed spacer sequences. Mycologia. 2016;108:1–5. 10.3852/14-293 26553774

[29] Edgar RC (2018). Accuracy of taxonomy prediction for 16S rRNA and fungal ITS sequences. PeerJ.

[30] Sipos B, Massingham T, Jordan GE, Goldman N (2011). PhyloSim -Monte Carlo simulation of sequence evolution in the R statistical computing environment. BMC Bioinformatics. BioMed Central Ltd.

[31] Claesson MJ, O'Sullivan O, Wang Q, Nikkilä J, Marchesi JR, Smidt H, et al. Comparative analysis of pyrosequencing and a phylogenetic microarray for exploring microbial community structures in the human distal intestine. Ahmed N, editor. PLoS One. 2009;4:e6669. 10.1371/journal.pone.0006669 PMC2725325 19693277

[32] Consortium THMP (2012). A framework for human microbiome research. Nature Nature Publishing Group.

[33] Fouhy F, Clooney AG, Stanton C, Claesson MJ, Cotter PD. 16S rRNA gene sequencing of mock microbial populations- impact of DNA extraction method, primer choice and sequencing platform. BMC Microbiol. 2016;16:123. 10.1186/s12866-016-0738-z PMC4921037 27342980

[34] Salter SJ, Cox MJ, Turek EM, Calus ST, Cookson WO, Moffatt MF (2014). Reagent and laboratory contamination can critically impact sequence-based microbiome analyses. BMC Biol.

[35] de Goffau MC, Lager S, Salter SJ, Wagner J, Kronbichler A, Charnock-Jones DS, et al. Recognizing the reagent microbiome. Nat Microbiol. 2018;3:851–3. 10.1038/s41564-018-0202-y 30046175

[36] Hahn MW, Jezberová J, Koll U, Saueressig-Beck T, Schmidt J. Complete ecological isolation and cryptic diversity in *Polynucleobacter* bacteria not resolved by 16S rRNA gene sequences. ISME J. 2016;10:1642–55. 10.1038/ismej.2015.237 PMC4913878 26943621

[37] Antony-Babu S, Stien D, Eparvier V, Parrot D, Tomasi S, Suzuki MT. Multiple *Streptomyces* species with distinct secondary metabolomes have identical 16S rRNA gene sequences. Sci Rep. 2017;7:11089. 10.1038/s41598-017-11363-1 PMC5593946 28894255

[38] Rosselló-Móra R, Amann R (2015). Past and future species definitions for Bacteria and Archaea. Syst Appl Microbiol.

[39] Segata N, Börnigen D, Morgan XC, Huttenhower C (2013). PhyloPhlAn is a new method for improved phylogenetic and taxonomic placement of microbes. Nat Commun.

[40] Abby SS, Tannier E, Gouy M, Daubin V (2012). Lateral gene transfer as a support for the tree of life. Proc Natl Acad Sci U S A.

[41] Parks DH, Chuvochina M, Waite DW, Rinke C, Skarshewski A, Chaumeil P-A, et al. A proposal for a standardized bacterial taxonomy based on genome phylogeny. bioRxiv. 2018;1:1–20. 10.1038/nbt.4229 30148503

