# Supplementary material for: IDTAXA: a novel approach for accurate taxonomic classification of microbiome sequences
Source: Microbiome. 2018 Aug 9;6:140. doi: 10.1186/s40168-018-0521-5 (PMC6085705; doi:10.1186/s40168-018-0521-5)
Supplement: Supplementary file 1 — Supplemental figures S1-S7. (PDF 764 kb) [file 40168_2018_521_MOESM1_ESM.pdf]

Additional file 1

Supplemental figures for Murali, A., Bhargava, A., & Wright, E. IDTAXA: a novel approach for accurate taxonomic classification of microbiome sequences. Microbiome (2018).

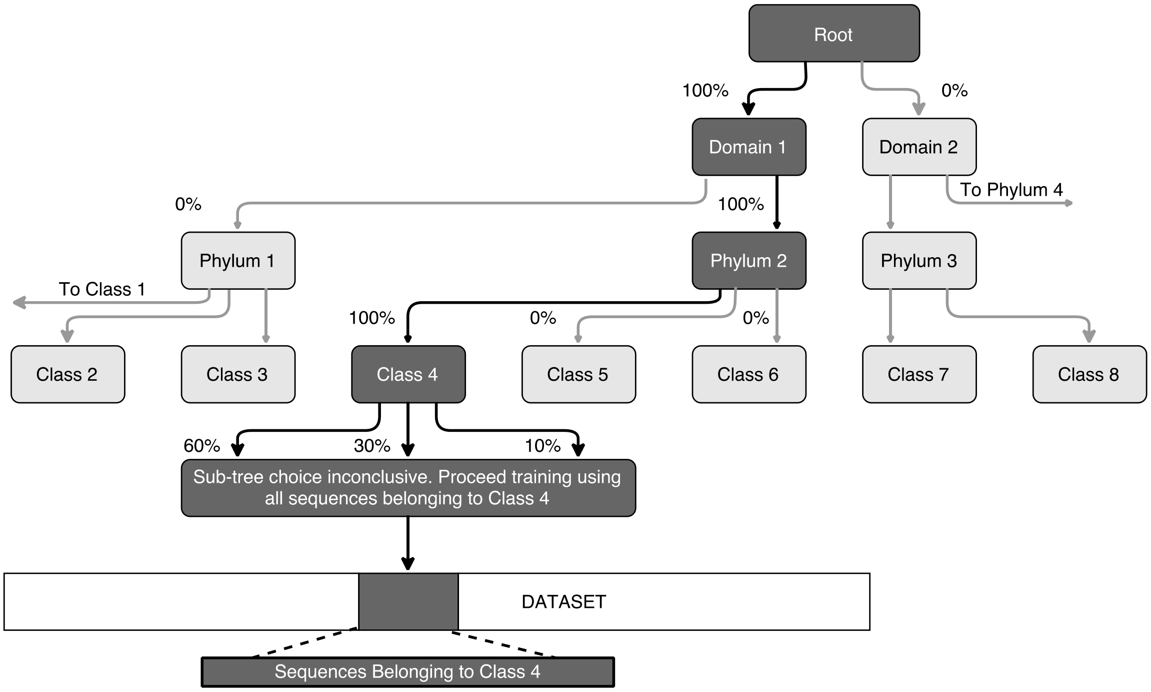

Figure S1. Schematic representation of the tree descent algorithm. In order to speed up the classification process, sequences are initially classified using a process termed "tree descent" that identifies the relevant subset of the training dataset to use for subsequent classification steps. At every rank level, bootstrap confidence is calculated for each taxonomic subgroup using the set of "decision k-mers" that best distinguish among alternative groups at that node. The taxonomic subgroup with highest confidence is selected and the tree is descended until confidence is less than 98% (by default) for any subgroup. Only sequences that are part of the training dataset below this node need to be considered in subsequent phases of the IDTAXA algorithm. Notably, the tree descent process is also used by LearnTaxa during the training phase to flag putative "problem sequences" and "problem groups" that may indicate labeling errors in the reference taxonomy.

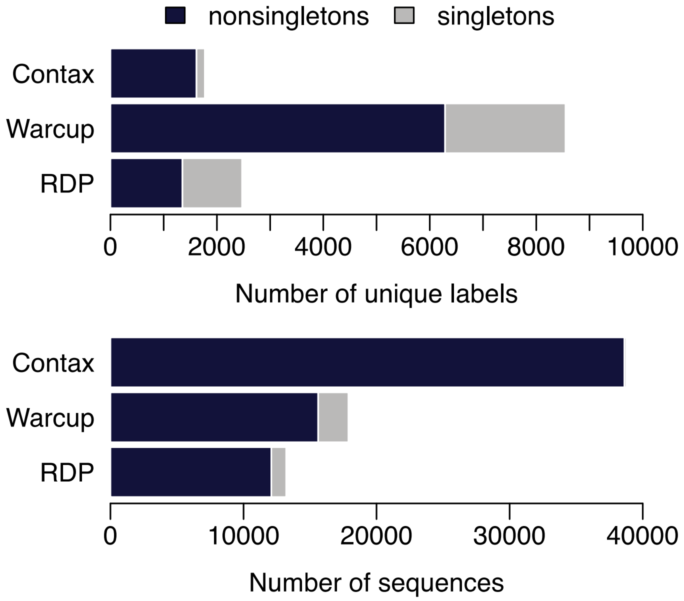

Figure S2. Comparison of different training sets used for benchmarking. The RDP (16S) and Warcup (ITS) training sets both contain many singletons, where a taxonomic group has only a single representative sequence. The Contax training set has the fewest labels but the most sequences on average per label.

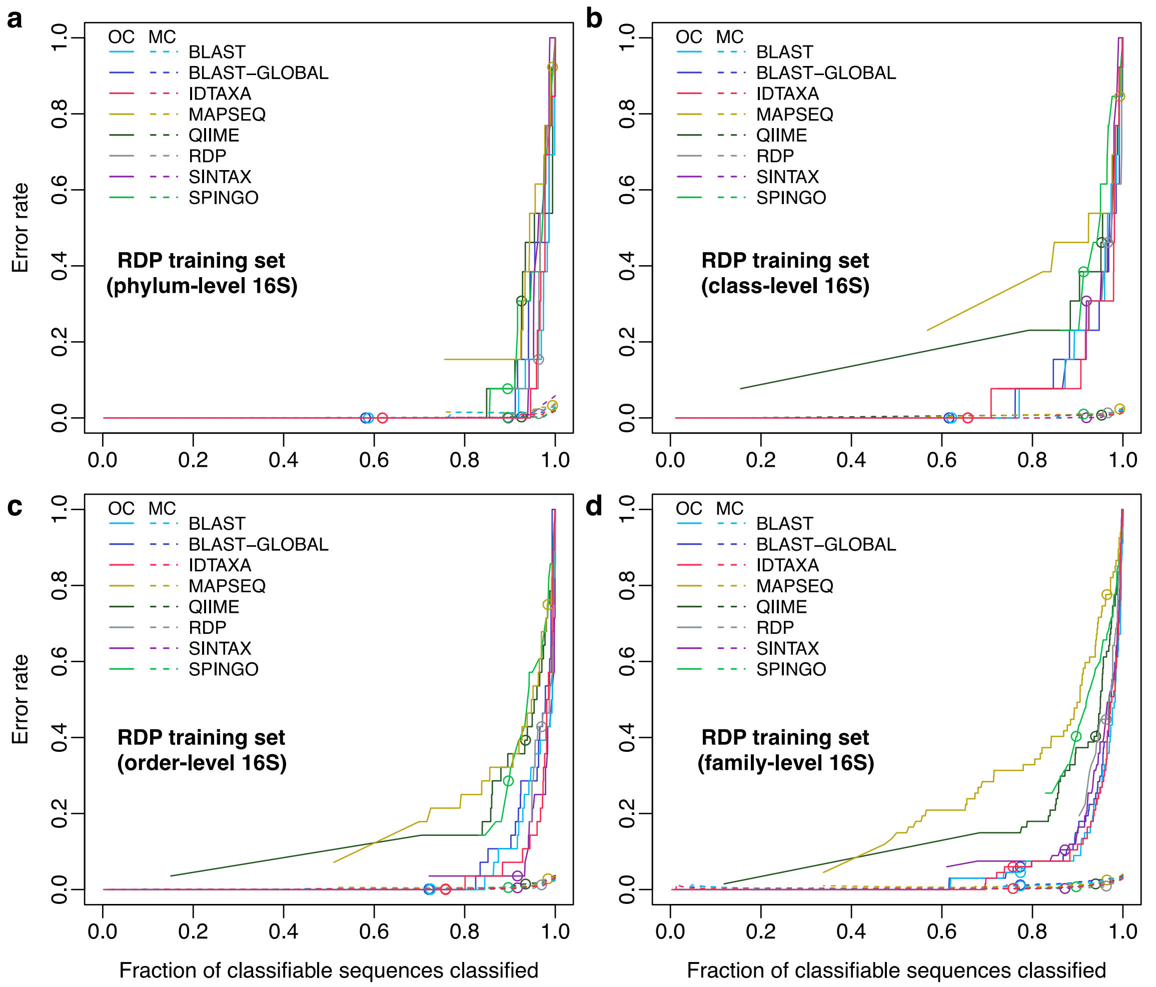

Figure S3. Error rates vary across rank levels. As expected, error rates during leave-one-out cross-validation are lower at the higher rank levels than they are for the basal (genus) rank (Fig. 1a) on the RDP training set. There are almost no errors at the phylum level (**a**), and only a few errors at the class (**b**), order (**c**), and family (**d**) levels. Note that the number of possible over classification (OC) and misclassification (MC) errors changes at different rank levels because the number of singleton groups differs.

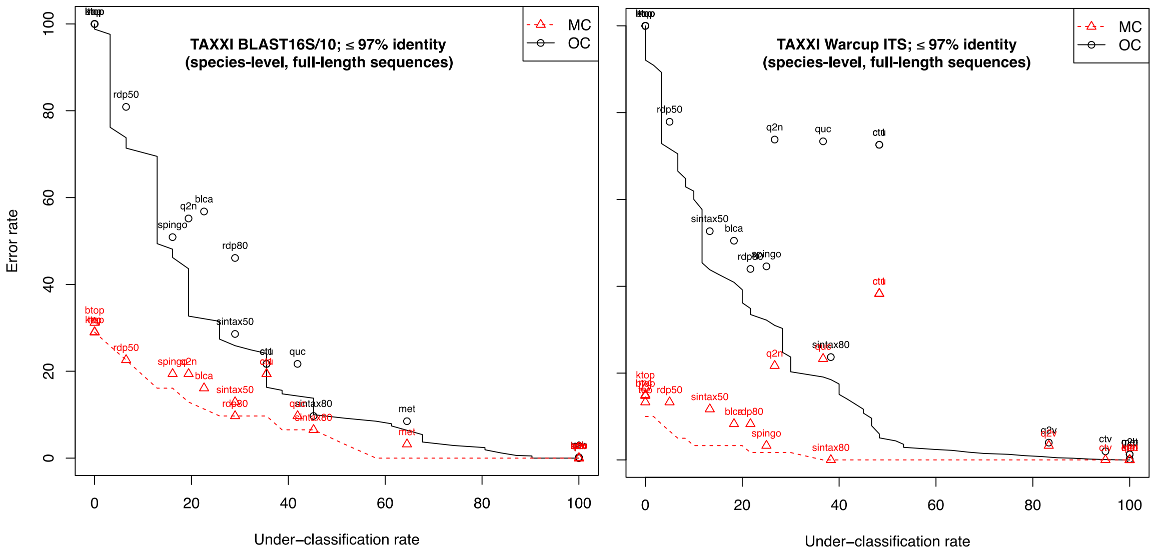

Figure S4. Accuracy on the independent TAXXI benchmark. Cross-validation by identity has been proposed as superior to leave-one-out cross-validation because it controls for the distance between the query sequences and their nearest training sequence [1]. Results are shown for the BLAST16S/10 (left) and Warcup ITS (right) full-length sequence test sets at the lowest rank level (species). Each benchmark is composed of test sequences that are at most 97% identical to any training (reference) sequence. IDTAXA (lines) displayed substantially lower over classification (OC) and misclassification (MC) error rates than any of the other classifiers tested (points). Points are labeled by program in accordance with the TAXXI benchmark [1]: BLCA (blca), BTOP (btop), CT1 (ctu), CT2 (ctv), KNN (knn), KTOP (ktop), Microclass (mc), Metataxa2 (met), NBC (nbc), Q2_BLAST (q2b), Q2_SK (q2n), Q2_VS (q2v), Q1 (quc), RDP at 50% confidence (rdp50), RDP at 80% confidence (rdp80), SINTAX at 50% confidence (sintax50), SINTAX at 80% confidence (sintax80), SPINGO (spingo), and TOP (top).

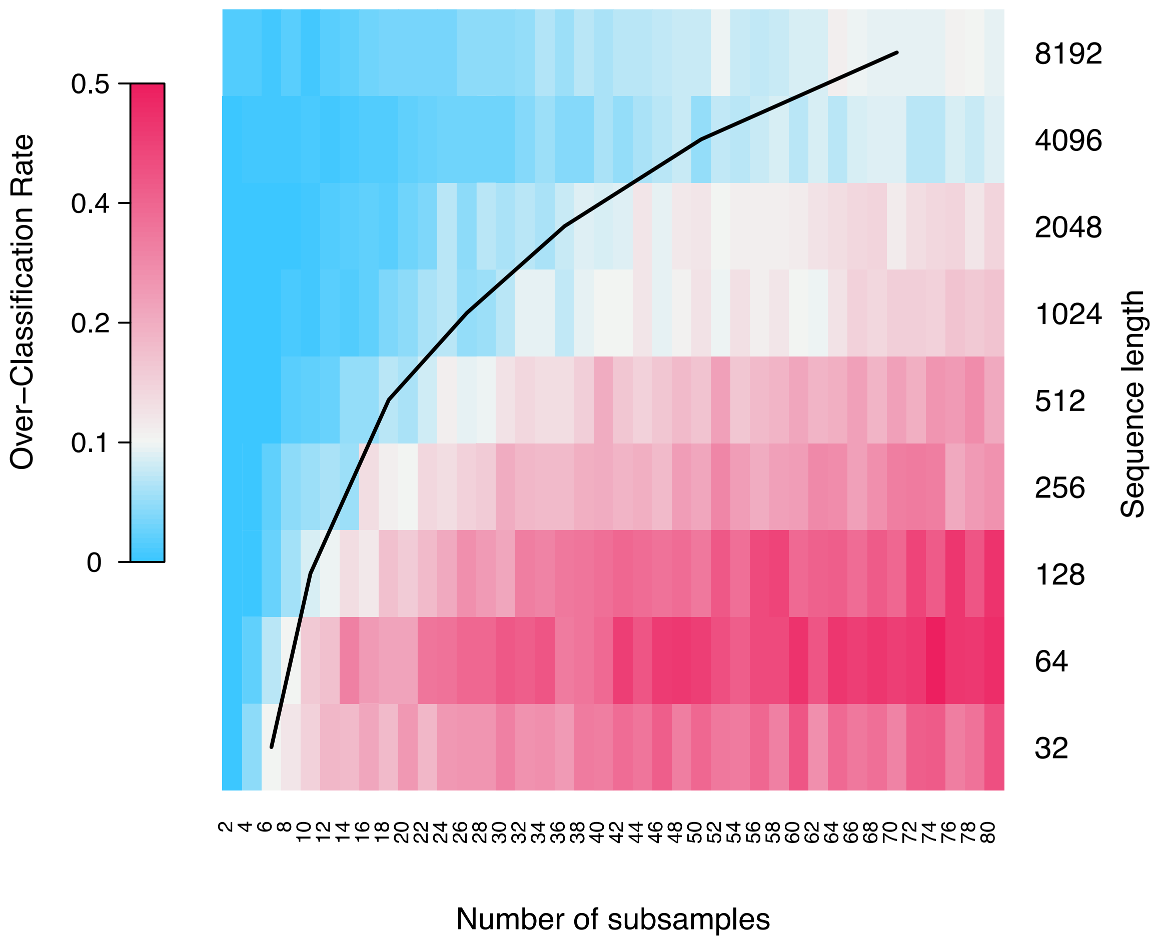

Figure S5. Optimizing the number of subsamples. Leave-one-out cross-validation was performed on a simulated training set of 1,000 sequences with varying lengths. The number of subsamples, *S*, required to keep the OC error rate roughly constant appeared to follow the function $S(l) = l^x$. A curve is shown for a value of *x* equal to 0.47.

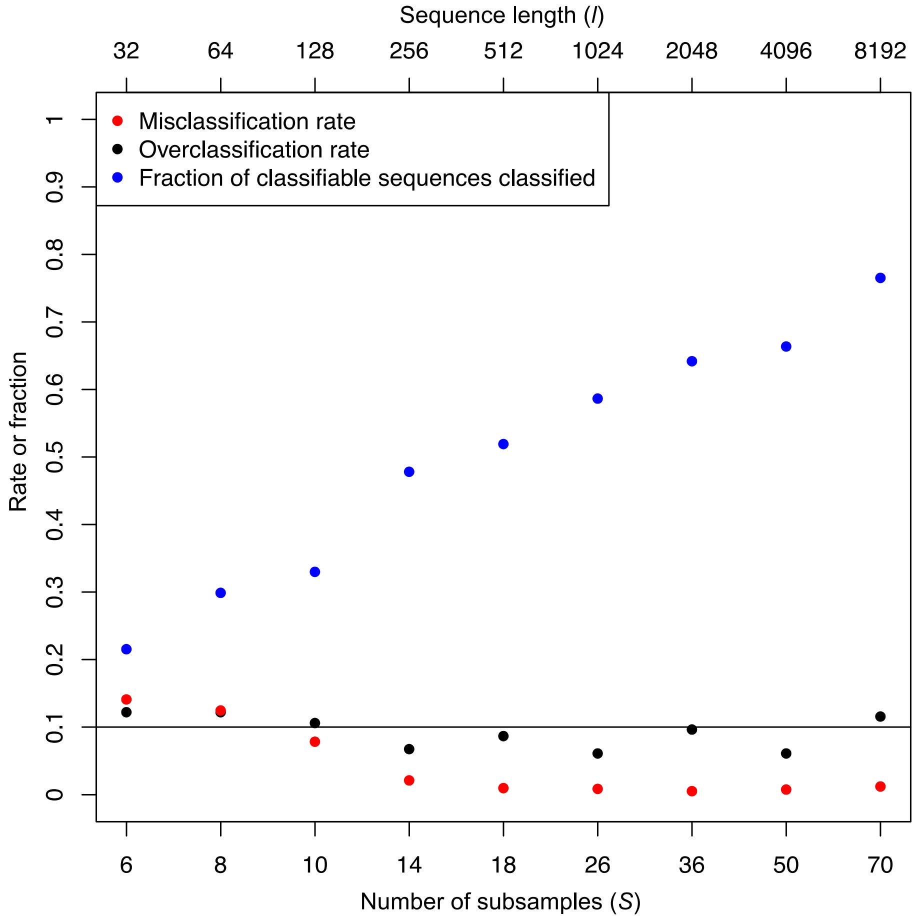

Figure S6. OC error rates are consistent across sequence lengths. Using the function $S(l) = l^{0.47}$ for selecting the optimal number of subsamples, OC error rates remain roughly constant (horizontal line) regardless of input query sequence length during leave-one-out classification on a simulated training set of 1,000 sequences with varying lengths. However, the MC error rate decreases and the fraction of classifiable sequences classified increases for longer sequence lengths.

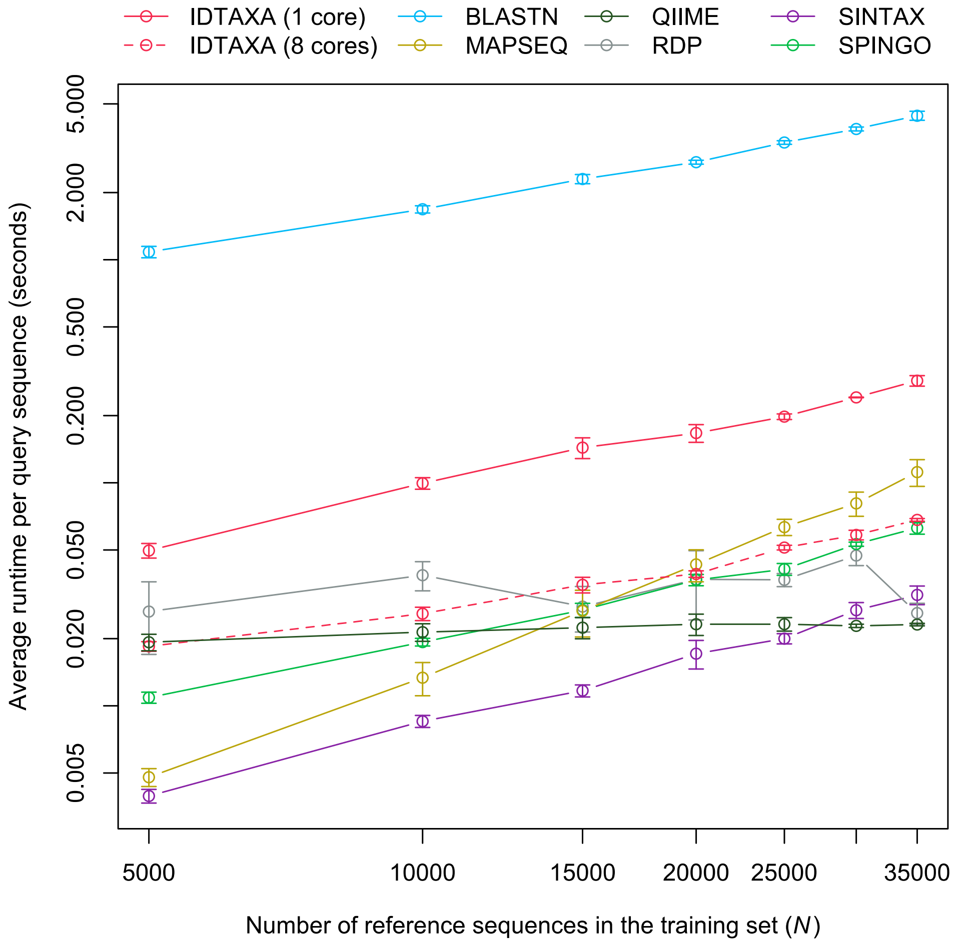

Figure S7. IDTAXA exhibits sub-linear scalability with reference size. The average runtime required to process each of 1,000 input query sequences is shown relative to the size (*N*) of the subset of the Contax training set used for training. IDTAXA was about 10-fold slower than the fastest classifier (SINTAX), but displayed about a 4-fold speedup when using 8 processor cores. BLAST was by far the slowest method, while the RDP classifier displayed the best scalability. Error bars show plus or minus one standard deviation from the mean of three replicates. Note the log-scaled axes.

References

1. Edgar RC. Accuracy of taxonomy prediction for 16S rRNA and fungal ITS sequences. PeerJ. 2018;6:e4652.
